# Clinical Determinants of Dose–Length Product in Pediatric Brain CT: Implications for Age-Specific Imaging Protocols

**DOI:** 10.3390/jimaging12080383

**Published:** 2026-08-14

**Authors:** Kangmin Lee, Jina Shim, Youngjin Lee

**Affiliations:** 1 Department of Radiology, Korea University Anam Hospital, Seoul 02841, Republic of Korea; kumc20132@kumc.or.kr; 2 Department of Radiological Science, Wonkwang University, Iksan 54538, Republic of Korea; 3 Department of Radiological Science, Gachon University, Incheon 21936, Republic of Korea

**Keywords:** pediatric brain CT, radiation dose, dose optimization, multiple linear regression analysis, age-related development

## Abstract

Systematic optimization of pediatric brain computed tomography (CT) protocols remains challenging because clinical data are limited for children younger than 5 years. Sixty-nine non-contrast brain CT examinations in children aged 5 years or younger were retrospectively analyzed. Multiple linear regression was used to assess associations between dose–length product (DLP) and age group, sex, body mass index (BMI), and scanner group. In univariable analyses, DLP differed significantly by age group (*p* < 0.001) and scanner group (*p* < 0.001). In the multivariable model, only age group remained significantly associated with DLP; examinations in children aged 1–5 years showed an adjusted DLP increase of 158.71 mGy·cm compared with examinations in children younger than 1 year (*p* < 0.001). BMI and scanner group were not independently associated with DLP. Model stability was supported by residual normality and absence of multicollinearity. This study found that age group was the only measured variable significantly associated with DLP after adjustment, whereas BMI, sex, and scanner group were not significant. These findings highlight the importance of considering age when interpreting dose variation in pediatric brain CT.

## 1. Introduction

Children are generally more sensitive to ionizing radiation than adults because of rapid growth, higher cell proliferation rates, and longer remaining life expectancy. Accordingly, adherence to the As Low As Reasonably Achievable (ALARA) principle is important for minimizing unnecessary radiation exposure while maintaining diagnostic image quality. Previous studies have suggested that children younger than 5 years, particularly those aged 0–3 years, may be more susceptible to radiation-related effects than older children and adults. Kutanzi et al. [1] reported that children younger than 5 years are at a significantly higher risk of cancer from identical radiation doses owing to immature DNA damage repair mechanisms and high cell proliferation rates. Early childhood is a period of active brain development, including hippocampal and white matter maturation. Radiation exposure during this period may be associated with developmental effects on neural progenitor cells and long-term neurocognitive outcomes [2]. Experimental studies in juvenile animal models have also reported hippocampal injury and neuroinflammatory changes after radiation exposure during early childhood [3]. The thyroid is also considered a radiosensitive organ during childhood. Thyroid cell proliferation is relatively active during early childhood, and radiation exposure during childhood has been associated with an increased risk of thyroid malignancy in epidemiological and radiation biology studies [3]. In addition, several cohort studies have reported increased risks of hematologic malignancies and brain tumors after pediatric CT exposure, with risk estimates generally higher among younger children [4]. However, the magnitude of risk varies across studies and exposure conditions [5,6]. These findings support careful justification and optimization of CT examinations in young children.

Despite the need for careful radiation dose management in children younger than 5 years, systematic optimization of pediatric brain CT protocols remains challenging because clinical imaging data in this age group are limited. Previous studies of body CT, including abdominal and chest CT, have reported associations between body mass index (BMI) and radiation dose indices in pediatric and adult populations [7,8,9]. These findings support patient-size-adjusted dose optimization. However, whether BMI, an anthropometric index related to body habitus, is similarly associated with dose modulation in pediatric brain CT remains unclear. Furthermore, a recent study suggested that macrocephaly may be associated with overgrowth syndromes that are occasionally accompanied by early-onset obesity or abnormal weight gain [10]. In pediatric brain CT, greater head size may influence attenuation on scout images and, consequently, automatic exposure control (AEC)-based tube current modulation. AEC is designed to adjust tube current according to patient attenuation and head size; therefore, increased scanner-reported dose indices may reflect size-based modulation rather than inappropriate dose delivery.

Therefore, this study evaluated factors associated with variation in scanner-reported DLP in non-contrast brain CT examinations of children aged 5 years or younger. Specifically, we examined whether age group, sex, BMI, and scanner group were independently associated with DLP. Given the limited clinical data available for pediatric brain CT in this age group, a particular objective was to determine whether BMI, which is commonly used as a patient-size surrogate in body CT, provides independent explanatory value for dose variation in pediatric brain CT after adjustment for age and scanner group. By addressing this question in a very young pediatric cohort, this study aimed to clarify the applicability of BMI to pediatric head CT dose assessment, distinguish patient-related variation from scanner- or protocol-related variation, and provide preliminary clinical evidence to guide future dose-optimization research.

## 2. Materials and Methods

### 2.1. Study Population and Data Collection

This retrospective cohort study included children aged 5 years or younger who underwent non-contrast brain CT at a single institution between January 2024 and June 2026. The study was approved by the Institutional Review Board of the participating institution, and the requirement for informed consent was waived due to the retrospective study design [IRB number: 2026AN0361].

**Figure 1:**
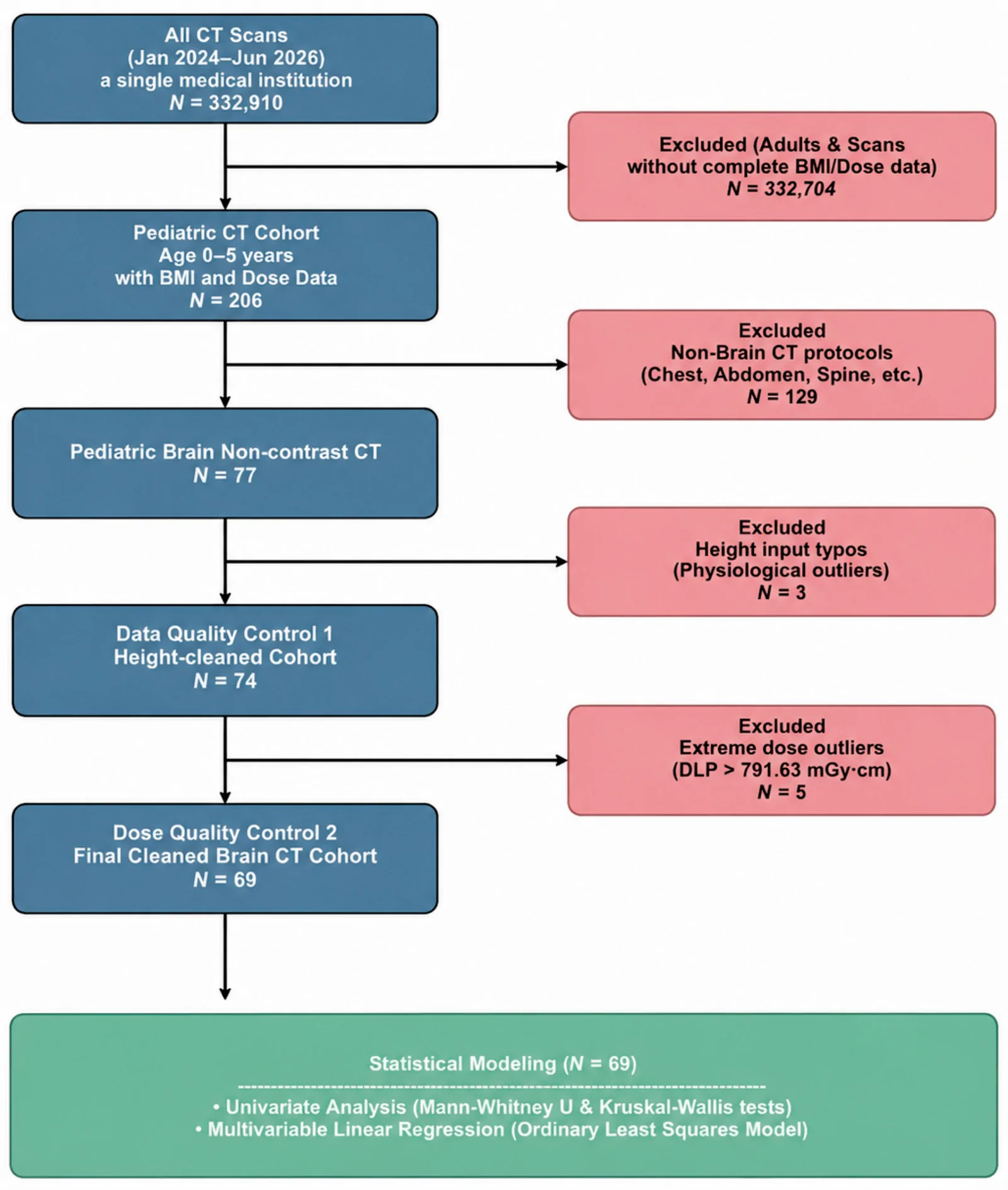
Schematic diagram of the study design and patient enrollment flow.

The study design and participant selection process are illustrated in Figure 1. In total, 332,910 CT examinations were performed using an institutional database. After excluding adult examinations and records with missing or incomplete BMI or dose information, 206 pediatric records were identified. Second, to establish a single clinical cohort, 129 examinations involving protocols other than brain imaging, such as chest, abdominal, and spine imaging, were excluded, resulting in a final dataset of 77 non-contrast brain CT examinations. Strict data quality control and outlier refinement criteria were applied to ensure robustness of the statistical analysis. Third, three records with implausible BMI values due to erroneous entry heights were excluded (*n* = 74). In the fourth step, five examinations with dose–length product (DLP) values exceeding the upper Tukey fence, defined as the third quartile plus 1.5 times the interquartile range, were excluded as statistical outliers. The corresponding cutoff value was 791.63 mGy·cm. The final analytical sample comprised 69 eligible brain CT scans.

Univariable and multivariable linear regression analyses were performed. To reduce instability in regression estimates caused by sparse observations within individual scanner groups, the seven CT scanners were grouped into four groups. Scanners with limited sample sizes were merged based on the data distribution to mitigate potential coefficient distortion from small subgroups. For multivariable analysis, the scanner positioned in the median range of the overall dose distribution was designated as the reference group.

In the final analytical sample, 35 examinations were performed in male patients (50.7%) and 34 in female patients (49.3%). The mean age was 1.26 years (range, 0–5 years). Mean height, weight, and BMI were 78.88 cm (range, 52.0–117.0 cm), 10.55 kg (range, 4.0–21.0 kg), and 16.44 kg/m^2^ (range, 12.1–28.5 kg/m^2^), respectively.

### 2.2. CT Scanner and Imaging Protocols

Non-contrast brain CT examinations were identified from institutional imaging metadata. Seven CT scanners were included in the assessment. Manufacturer, model, detector configuration, tube voltage, tube current modulation method, rotation time, pitch, slice thickness, and reconstruction algorithm were reviewed for each scanner.

All examinations were performed according to the institutional non-contrast brain CT protocol, and the analysis was restricted to non-contrast CT examinations of the head to ensure anatomical consistency. Consequently, only pediatric non-contrast brain CT scans were included in the study cohort.

The imaging parameters of each scanner were configured according to the institutional pediatric brain CT protocol. Tube voltage was set within the range of 70–100 kVp or 80–100 kVp, depending on the specific equipment and protocol. Automatic tube current modulation (ATCM) was applied using proprietary methods for each scanner, including CARE Dose4D, Auto mA + SmartmA + ASiR-V, and DoseRight ACS. Acquired images were reconstructed to a slice thickness of 1 mm using scanner-specific iterative reconstruction algorithms (SAFIRE, ADMIRE, ASiR, and iDose4). The dose–length product (DLP), automatically recorded by each CT scanner after examination, was used as the primary dependent variable because it reflects both the scan range and radiation dose calculated by the equipment and represents a scanner-reported dose index rather than a direct measure of individual absorbed dose.

To address imbalance in sample size across scanners, seven scanners were initially identified as separate models. Scanners with adequate sample sizes—Scanner A (SOMATOM Definition Flash; Siemens Healthineers, Forchheim, Germany), Scanner B (SOMATOM Force; Siemens Healthineers, Forchheim, Germany), and Scanner C (Revolution CT ES; GE Medical Systems, Waukesha, WI, USA)—were retained as individual categories, whereas scanners with limited observations were combined into the “Other” (Ingenuity CT, IQon Spectral CT, Spectral CT 7500; Philips Healthcare, Best, The Netherlands) category to reduce unstable regression estimates. The final regression analysis therefore included four scanner groups: Scanner A, Scanner B, Scanner C, and Other. Scanner A was used as the reference category because its DLP distribution was near the center of the observed range. Although the scanners included in the Other category were operated under the institutional pediatric brain CT protocol and used DoseRight ACS and iDose4, they differed in model and technical specifications. Therefore, this grouping was made solely to improve statistical stability and should not be interpreted as evidence of technical equivalence among the scanners. Detailed scanner models and acquisition parameters are presented in Table 1.

### 2.3. Statistical Analysis

The distribution of DLP was assessed using the Shapiro–Wilk test. As the DLP values were not normally distributed, non-parametric tests were used for univariable group comparisons. The Mann–Whitney U test was used to compare DLP distributions between two independent groups, including sex (male vs. female) and age (younger than 1 year vs. 1–5 years). The Kruskal–Wallis test was used to compare DLP distributions across three descriptive BMI ranges (≤15.0 kg/m^2^, >15.0 to ≤16.0 kg/m^2^, and >16.0 kg/m^2^) and across the four scanner groups. These ranges were used only for the descriptive univariable comparison and were not intended to represent clinical pediatric weight-status categories. BMI was entered as a continuous variable in the multivariable regression model. When the Kruskal–Wallis test showed a statistically significant difference, post hoc pairwise comparisons were performed using an appropriate multiple-comparison adjustment (Bonferroni correction). Continuous variables were summarized as means with ranges or medians with interquartile ranges, as appropriate. Regression coefficients and their 95% confidence intervals were reported for the multivariable analyses.

Multiple linear regression analysis was performed to estimate associations between DLP and age group, sex, BMI, and scanner group after adjustment for the other covariates. The reference categories were female sex, the group younger than 1 year, and Scanner A. Model performance was evaluated using the coefficient of determination (*R*^2^), adjusted *R*^2^, Akaike information criterion (AIC), and F-statistic.

Multicollinearity among the independent variables in the multivariable regression model was assessed using the variance inflation factor (VIF), and adherence to the conventional threshold of VIF < 5 was verified. The regression assumptions were evaluated using residual analysis. Residual normality was assessed using the Jarque–Bera and Shapiro–Wilk tests, and residual plots were reviewed to evaluate linearity, homoscedasticity, and influential observations.

To assess whether the association between BMI and DLP differed across the scanner groups, an extended regression model including a BMI × scanner group interaction term was fitted. The base and extended models were compared using an F-test, and the final model was selected based on the model fit and statistical significance. Statistical significance was set at *p* < 0.05. All statistical analyses were performed using SPSS Statistics version 27.0 (IBM Corp., Armonk, NY, USA).

## 3. Results

### 3.1. Data Refinement

**Figure 2:**
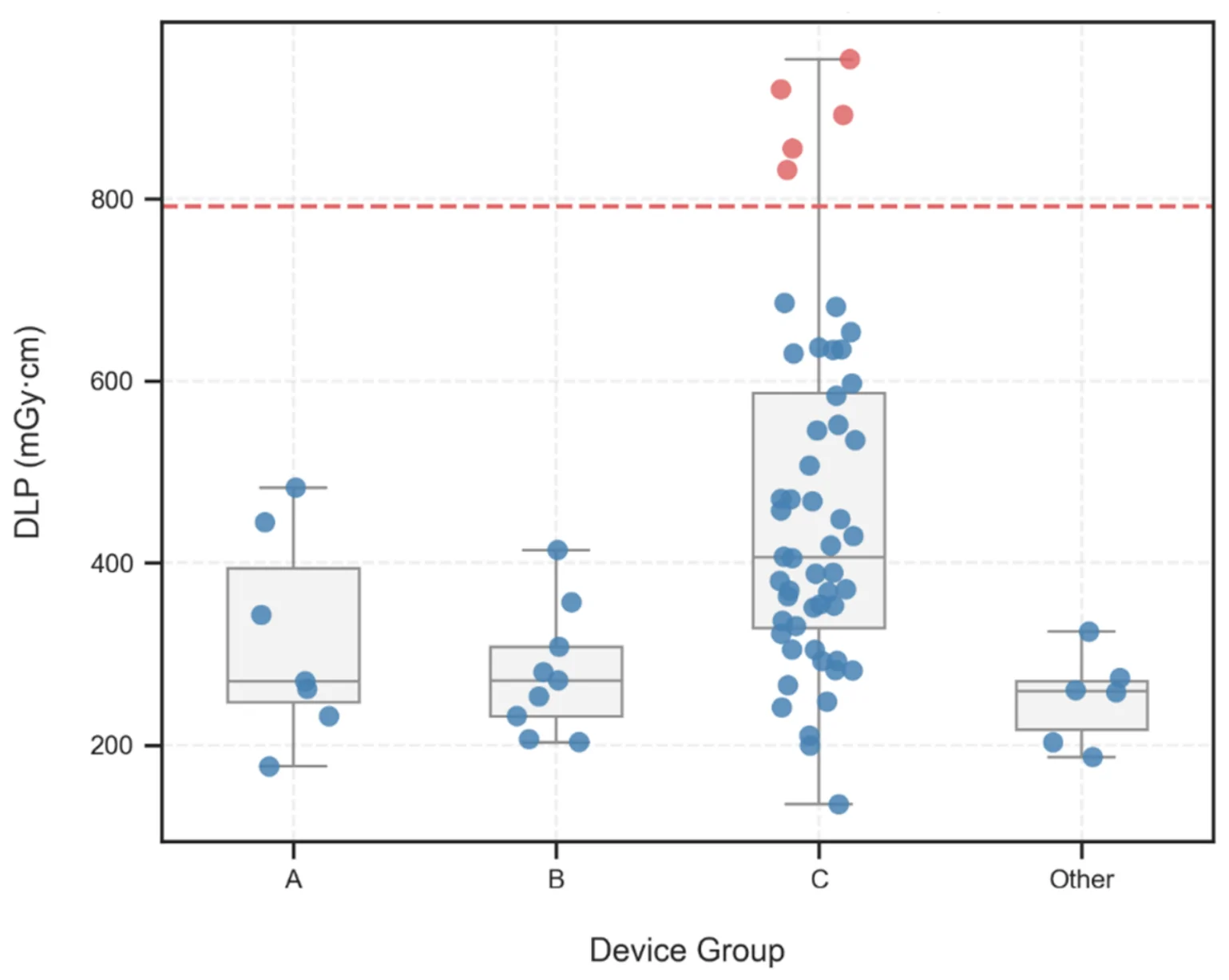
Dose outlier identification across CT scanner groups. The box-and-whisker plot with a jittered strip plot shows the distribution of dose–length product across the four CT scanner groups for *n* = 74 pediatric brain CT examinations. The gray boxes represent the interquartile range (IQR), and the solid horizontal line inside each box shows the median value. Blue circles indicate normal dose levels, whereas red circles represent the five extreme dose outliers exceeding the upper IQR threshold, shown by the red dashed line.

A total of 77 noncontrast brain CT examinations in children aged 5 years or younger were initially identified. Following a predefined data preprocessing procedure, three records with implausible BMI values attributable to erroneous height entries were excluded. An outlier criterion based on the interquartile range (IQR) of the DLP values within the brain CT cohort was applied. Five examinations with DLP values exceeding the upper Tukey fence of 791.63 mGy·cm were identified as statistical outliers and excluded. All five excluded outliers were observed in examinations performed using Scanner C, and three of the five examinations involved patients younger than 1 year (Figure 2). The final analytic sample comprised 69 examinations.

The seven CT scanners were subsequently classified into four groups: Scanner A, Scanner B, Scanner C, and Other. Scanner A (*n* = 7), with a mean DLP of 315.51 mGy·cm, was selected as the reference group because its mean DLP was near the center of the observed scanner-level distribution.

### 3.2. Univariable Analysis

**Figure 3:**
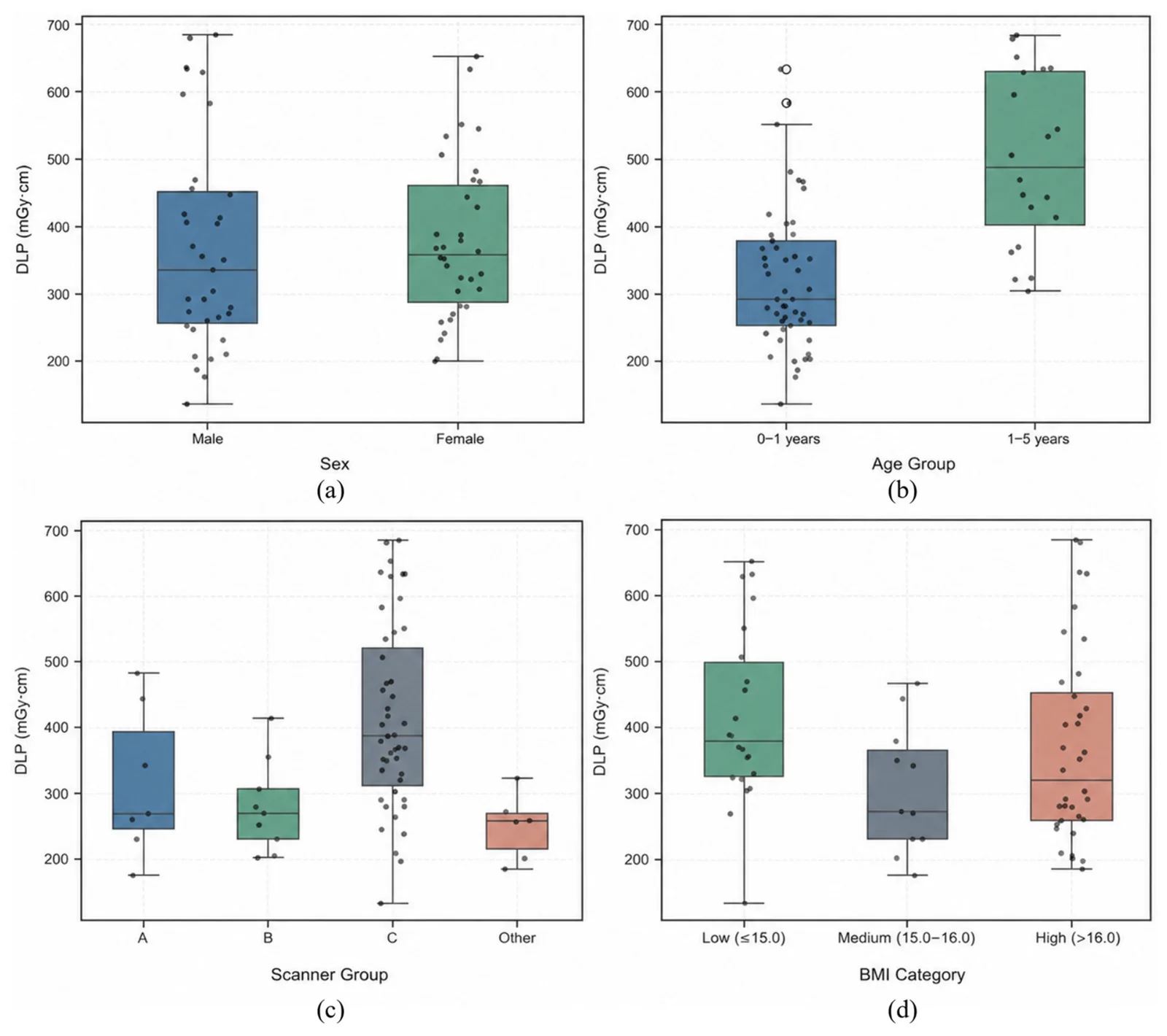
Univariable comparisons of dose–length product (DLP) across clinical and scanner factors. Box-and-whisker plots compare DLP across clinical and scanner factors in the cleaned cohort (*n* = 69). Individual patient data are plotted as black jittered dots to show sample density. Subgroup sample sizes are displayed directly in the *x*-axis labels. (**a**) DLP by sex: male (*n* = 35) and female (n = 34); (**b**) DLP by age group: younger than 1 year (*n* = 32) and 1–5 years (*n* = 37); (**c**) DLP by scanner group: Scanner A (*n* = 7), Scanner B (*n* = 9), Scanner C (*n* = 47), and Other (*n* = 6); and (**d**) DLP by descriptive BMI range: ≤15.0 kg/m^2^ (*n* = 22), >15.0 to ≤16.0 kg/m^2^ (*n* = 11), and >16.0 kg/m^2^ (*n* = 36).

The Shapiro–Wilk test indicated that DLP values were not normally distributed (*p* = 0.003); therefore, nonparametric tests were used for group comparisons. DLP distributions differed significantly between the age groups. The 1–5-year group showed higher DLP values than the group younger than 1 year, with an observed difference in the reported central tendency of 177.01 mGy·cm (*p* < 0.001; Figure 3b). DLP distributions also differed significantly among scanner groups (Kruskal–Wallis *p* < 0.001). Post hoc pairwise comparisons indicated that Scanner C had significantly higher DLP values than Scanner B (*p* = 0.0108; Figure 3c). No statistically significant differences in DLP distribution were observed according to sex (*p* = 0.4977) or BMI category (*p* = 0.0576; Figure 3a,d).

### 3.3. Multiple Linear Regression Analysis

**Figure 4:**
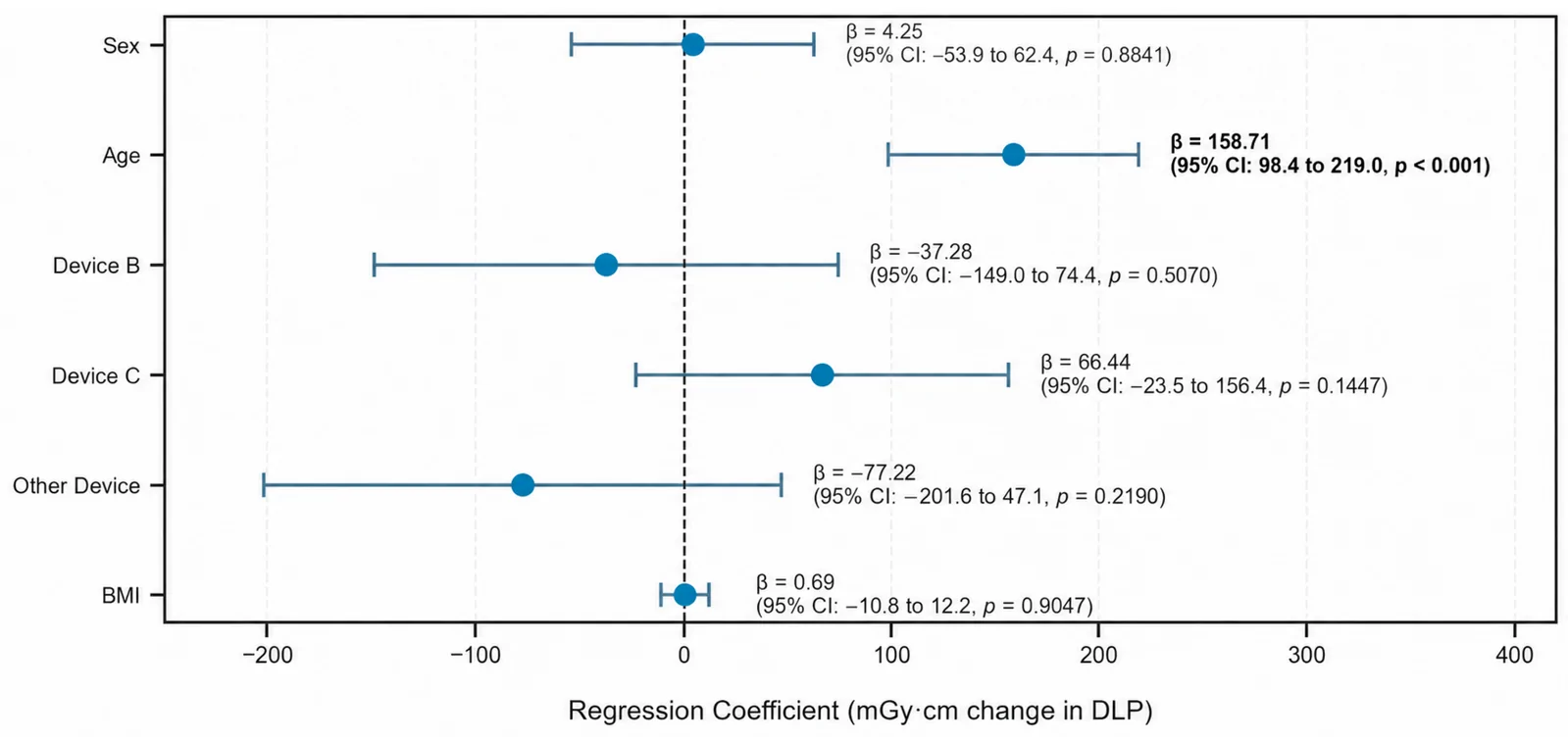
Forest plot of regression coefficients for clinical and scanner factors. The forest plot shows the multivariable regression coefficients and their 95% confidence intervals (error bars) from the ordinary least squares model. The reference categories are female sex, the group younger than 1 year, and Scanner A. Blue circles indicate estimated coefficients, error bars represent 95% confidence intervals, and the vertical dashed line indicates the null effect line.

Multiple linear regression analysis was performed to evaluate associations between DLP and age group, sex, BMI, and scanner group. The model had an *R*^2^ value of 0.46 and an adjusted *R*^2^ of 0.3986, indicating that the included covariates accounted for approximately 46.05% of the observed variation in DLP. The overall model was statistically significant (F-test *p* = 1.88 × 10^−6^), and the AIC was 849.25. The reference categories were female sex, the group younger than 1 year, and Scanner A. The detailed regression results are presented in Table 2 and Figure 4. As shown in Table 2, age was the only variable that showed a statistically significant association with DLP. After adjustment for sex, BMI, and scanner group, examinations in children aged 1–5 years were associated with an adjusted increase in DLP of 158.71 mGy·cm compared with examinations in children younger than 1 year (*p* < 0.001). No statistically significant associations with DLP were observed for sex, scanner group, or BMI in the multivariable model. The regression coefficient for male sex was 4.25 mGy·cm (*p* = 0.8841). None of the scanner groups differed significantly from the reference group, Scanner A (Scanner B, *p* = 0.5070; Scanner C, *p* = 0.1447; Other, *p* = 0.2190). Although Scanner C showed significantly higher DLP values than Scanner B in the univariable analysis, this difference was not statistically significant after multivariable adjustment. BMI was also not significantly associated with DLP (coefficient = 0.69 mGy·cm per kg/m^2^, *p* = 0.9047).

Multicollinearity was assessed using variance inflation factors (VIFs). VIF values were 1.25 for sex, 1.11 for age group, 1.82–2.61 for scanner group, and 1.33 for BMI [S1]. Residual normality was assessed using Jarque–Bera and Shapiro–Wilk tests. The Jarque–Bera test yielded *p* = 0.8815 and the Shapiro–Wilk test yielded W = 0.9892 and *p* = 0.8206, providing no evidence against the normality of the model residuals.

To investigate the interaction between BMI and scanner group, an interaction term (BMI × scanner group) was added to the model. The model yielded an $R^{2}$ of 0.47, an adjusted $R^{2}$ of 0.38, and an AIC of 854.02. Table 3 presents the results of additional analyses, including the interaction term. The regression coefficients and *p* values for the interaction terms were as follows: BMI × Scanner B, −37.84 (*p* = 0.3228); BMI × Scanner C, −20.57 (*p* = 0.5013); and BMI × Other, −18.49 (*p* = 0.6317). None of the interaction terms was statistically significant. Furthermore, an F-test evaluating the improvement in the model’s explanatory power after the inclusion of these terms yielded p = 0.7925, indicating no significant improvement in the extended regression model compared with the baseline model.

## 4. Discussion

Children are generally more sensitive to ionizing radiation than adults because of their developmental characteristics and longer life expectancies. Therefore, the ALARA principle is important when the CT findings are clinically justified. Previous epidemiological studies have reported associations between CT exposure during childhood and increased risks of leukemia and selected solid tumors [11,12,13]. In clinical practice, unnecessary CT examinations are avoided when alternative imaging modalities such as ultrasonography or magnetic resonance imaging can provide the required diagnostic information. However, because brain CT data in very young children are limited, the systematic evaluation of dose variation in this population remains challenging.

Factors associated with variation in scanner-reported DLP were evaluated in children aged 5 years or younger who underwent non-contrast brain CT. The analysis was specifically designed to determine whether BMI, a widely used determinant of radiation dose in body CT, also associated with dose modulation in brain CT. Macrocephaly may occur in some overgrowth syndromes and may coexist with increased body size in selected conditions [10]. AEC systems in CT scanners adjust tube current according to subject attenuation and size to maintain image quality [14,15]. Consequently, increased pediatric head circumference related to abnormal weight gain may be associated with mechanical compensatory mechanisms and a higher scanner-reported dose. This raises the clinical concern that pediatric patients may be exposed to a higher radiation dose without additional diagnostic benefit. Therefore, a multivariable model was used to clarify whether dose fluctuations during pediatric head CT were attributable to increased body habitus associated with elevated BMI or to skull development associated with chronological age.

During data refinement, five examinations with DLP values above the upper Tukey fence were excluded from the primary analysis. All five examinations were performed using Scanner C, and three involved patients younger than 1 year. BMI values in these examinations ranged from 16.0 to 18.4 kg/m^2^. Review of the available dose reports for all three examinations involving patients younger than 1 year showed that image acquisition was interrupted during the examination and subsequently resumed. This pattern suggests that repeat acquisition, possibly due to patient motion, may have contributed to the elevated cumulative DLP values. Because this retrospective dataset did not include direct measurements of head circumference, effective diameter, motion, positioning quality, sedation status, or detailed scanner logs, the reasons for the elevated DLP values could not be determined. The clustering of these outliers in one scanner group may reflect scanner-specific protocol characteristics, patient positioning, motion, patient factors, or other unmeasured variables. Considering that three of the five outliers occurred in patients younger than 1 year, factors common in pediatric examinations, such as motion, difficulty maintaining posture, and positioning variation, may have partially contributed to the increase in DLP.

From a statistical perspective, these five cases corresponded to outliers exceeding the upper limit of the overall DLP distribution, and their inclusion in the primary regression analysis could have biased estimation of factors associated with radiation dose in the general pediatric brain CT population. Therefore, these cases were excluded from the primary analysis to evaluate typical dose variation factors. However, these cases suggest that dose management in pediatric non-contrast brain CT is associated not only with patient physique or age but also with equipment-specific protocol operation and examination procedures. Consequently, these findings support periodic monitoring of equipment-specific dose distributions and consistent positioning and protocol application during infant examinations in pediatric CT.

Multiple regression analysis showed that age group, rather than BMI, was the only independent variable significantly associated with pediatric brain CT radiation dose. The 1–5-year group showed a significantly higher adjusted DLP of 158.71 mGy·cm compared with the group younger than 1 year. This result indicates an association between age group and scanner-reported DLP in this cohort but does not establish the underlying anatomical mechanism. Age-related changes in head diameter, skull thickness, ossification, and fontanelle status are plausible explanations, but none of these variables were directly measured. The nonsignificant BMI coefficient likewise should not be interpreted as evidence that body habitus has no influence; rather, BMI may be an imprecise surrogate for dose-relevant cranial dimensions in young children, and the limited sample size may have prevented detection of smaller effects. However, as detailed in Table 1, the scanners differed in tube-voltage range, AEC algorithm, rotation time, pitch, detector configuration, and iterative reconstruction method. Although scanner-specific protocols were implemented at the same institution with the clinical aim of obtaining diagnostically acceptable images, vendor-specific AEC and iterative reconstruction technologies may allow this clinical objective to be achieved at different radiation dose levels. These technical differences may also have influenced tube-current selection, scan duration, and scanner-reported DLP and therefore remain important considerations in dose variation and optimization. Therefore, the nonsignificant scanner coefficients should not be interpreted as evidence of technical equivalence, particularly given the limited scanner-specific sample sizes and the grouping of heterogeneous models within the Other category. The stability of these statistical inferences was supported by the absence of multicollinearity (VIF < 3) and residual normality (Shapiro–Wilk p=0.8206).

These results contrast with those of previous body CT studies and have clinical implications. Numerous studies have shown that patient BMI is positively correlated with the $CTDI_{vol}$ and DLP in pediatric and adult abdominal and thoracic CT [16,17], supporting dose protocol optimization based on BMI. In contrast, BMI was not significantly associated with DLP in this brain CT cohort. Whereas AEC algorithms in body CT detect body fat and soft tissue thickness from scout images to modulate dose [18,19], X-ray attenuation in head CT may be influenced by the composition and thickness of tissues within the cranial region, including osseous structures; therefore, BMI may have limited influence on AEC operation. BMI reflects whole-body habitus and may not accurately represent the size or attenuation characteristics of the head. From a physical perspective, X-ray attenuation is determined more directly by the composition and thickness of the tissues along the beam path. Compared with the body, the pediatric head has a smaller cross-sectional diameter, while high-attenuation osseous structures occupy a relatively substantial proportion of the scanned region. Therefore, variations in head diameter, skull thickness, ossification, and fontanelle status may influence total attenuation and AEC-selected tube current more directly than BMI. These physical characteristics may help explain why BMI was not significantly associated with DLP in the present study and suggest that direct cranial measurements may be more appropriate than whole-body BMI for pediatric head CT dose assessment and optimization. However, these anatomical variables were not directly measured in this study and should be evaluated in future research.

This study had some limitations. First, this was a retrospective single-center study, and the findings may not be generalizable to other institutions with different scanners, protocols, and patient populations. Second, the final sample size was limited to 69 examinations, with imbalanced subgroup sizes. A post hoc sensitivity analysis based on six predictor degrees of freedom, α = 0.05, and 80% power yielded approximate minimum detectable effect sizes of Cohen’s f^2^ = 0.12 for a one-degree-of-freedom predictor and f^2^ = 0.17 for the three-degree-of-freedom scanner-group term. These values indicate that the study was primarily capable of detecting effects approaching a moderate magnitude, whereas smaller associations may not have been detected reliably. Therefore, smaller associations, particularly those related to BMI and scanner group, may not have been detected, and nonsignificant findings should not be interpreted as evidence of no association. Third, age was dichotomized as younger than 1 year versus 1–5 years because the numbers of examinations at individual yearly ages were insufficient for stable multivariable estimates. This categorization was selected to distinguish infancy from the later early-childhood period; however, it did not allow assessment of age-related variation within the 1–5-year group or of a potentially nonlinear association between age and DLP. Fourth, although the five high-DLP examinations were excluded because they could disproportionately influence the evaluation of typical DLP variation, a formal sensitivity analysis including these observations was not performed. Therefore, their potential influence on the estimated DLP distribution, scanner comparison, and regression coefficients cannot be completely excluded. Fifth, this study evaluated scanner-reported dose variation but did not assess objective image noise, contrast-to-noise ratio, artifacts, or radiologist-rated diagnostic acceptability. Consequently, the observed DLP differences cannot determine whether the protocols achieved an optimal balance between radiation dose and image quality or whether lower-dose examinations remained diagnostically adequate. Finally, the most important limitation was that actual head circumference or effective head diameter, which are direct determinants of head radiation dose, could not be measured or included in the analysis. Despite these limitations, this study provides preliminary data regarding factors associated with DLP variation in brain CT examinations of children aged 5 years or younger. Accordingly, the present findings should be regarded as hypothesis-generating and require confirmation in larger prospective cohorts. Future studies should incorporate direct head-size measurements, size-specific dose estimates, detailed protocol variables, and diagnostic image quality assessments.

## 5. Conclusions

This study evaluated factors associated with DLP variation in non-contrast brain CT examinations in children aged 5 years or younger. After adjustment for sex, BMI, and scanner group, age group remained significantly associated with DLP, with examinations in children aged 1–5 years showing higher adjusted DLP values than those in children younger than 1 year. BMI, sex, scanner group, and BMI × scanner group interaction terms were not statistically significant in the adjusted model.

These findings suggest that age-related anatomical characteristics and protocol factors should be considered when DLP variation is interpreted in pediatric brain CT. These results should not be interpreted as evidence that BMI or scanner characteristics are irrelevant to dose modulation; rather, they indicate that no statistically significant associations were observed in this cohort after adjustment. Similarly, lower DLP should not be interpreted as superior protocol optimization without confirmation that diagnostic image quality is maintained. Further studies incorporating direct measurements of head size and additional technical variables and diagnostic image-quality assessment are needed to clarify the determinants of dose variation and to support pediatric brain CT dose optimization.

## Figures and Tables

**Table 1:** Detailed specifications and scan parameters of the CT scanners used for pediatric brain CT examinations.

Parameters	Scanner A	Scanner B	Scanner C	Other
Model name	Definition Flash	SOMATOM Force	Revolution CT ES	Ingenuity, IQon Spectral CT, Spectral CT 7500
Detector rows	128 (256 slices)	192 (384 slices)	256 (0.625 mm × 256)	64 (dual-layer spectral/128 slices)
kVp	80–100	70–100	70–100	80–100
Automatic exposure control	CARE Dose4D	CARE Dose4D	Auto mA + SmartmA + ASiR-V	DoseRight ACS
Rotation time (s)	1.0	1.0	0.7	0.33–0.4
Pitch	0.75	0.55	0.516	0.296–0.392
Slice thickness (mm)	1	1	1	1
Reconstruction	SAFIRE	ADMIRE	ASiR	iDose4

**Table 2:** Multiple linear regression analysis of factors affecting radiation dose, measured as dose–length product (DLP) in pediatric brain CT.

Variable	Coefficient	95% CI Lower	95% CI Upper	*p*-Value
Sex [Male]	4.25	−53.87	62.38	0.8841
Age group [1–5 years]	158.71	98.42	219.00	<0.001
Scanner [B]	−37.28	−148.98	74.42	0.5070
Scanner [C]	66.44	−23.49	156.36	0.1447
Scanner [Other]	−77.22	−201.56	47.11	0.2190
BMI	0.69	−10.79	12.17	0.9047

**Table 3:** Regression coefficients for BMI × scanner group interaction terms.

Variable	Coefficient	95% CI Lower	95% CI Upper	*p*-Value
BMI × Scanner [B]	−37.84	−113.79	38.12	0.3228
BMI × Scanner [C]	−20.57	−81.42	40.28	0.5013
BMI × Scanner [Other]	−18.49	−95.29	58.31	0.6317

## Data Availability

The original contributions presented in this study are included in the article. Further inquiries can be directed to the corresponding authors.

## Abbreviations

The following abbreviations are used in this manuscript:

CT	Computed tomography
DLP	Dose–length product
BMI	Body mass index
ALARA	As low as reasonably achievable
ATCM	Automatic tube current modulation
AIC	Akaike information criterion
VIF	Variance inflation factor
IQR	Interquartile range
AEC	Automatic exposure control
